# Supplementary figures and images for: Performance of self-reported measures of alcohol use and of harmful drinking patterns against ethyl glucuronide hair testing among young Swiss men
Source: PLoS One. 2020 Dec 23;15(12):e0244336. doi: 10.1371/journal.pone.0244336 (PMC7757898; doi:10.1371/journal.pone.0244336)

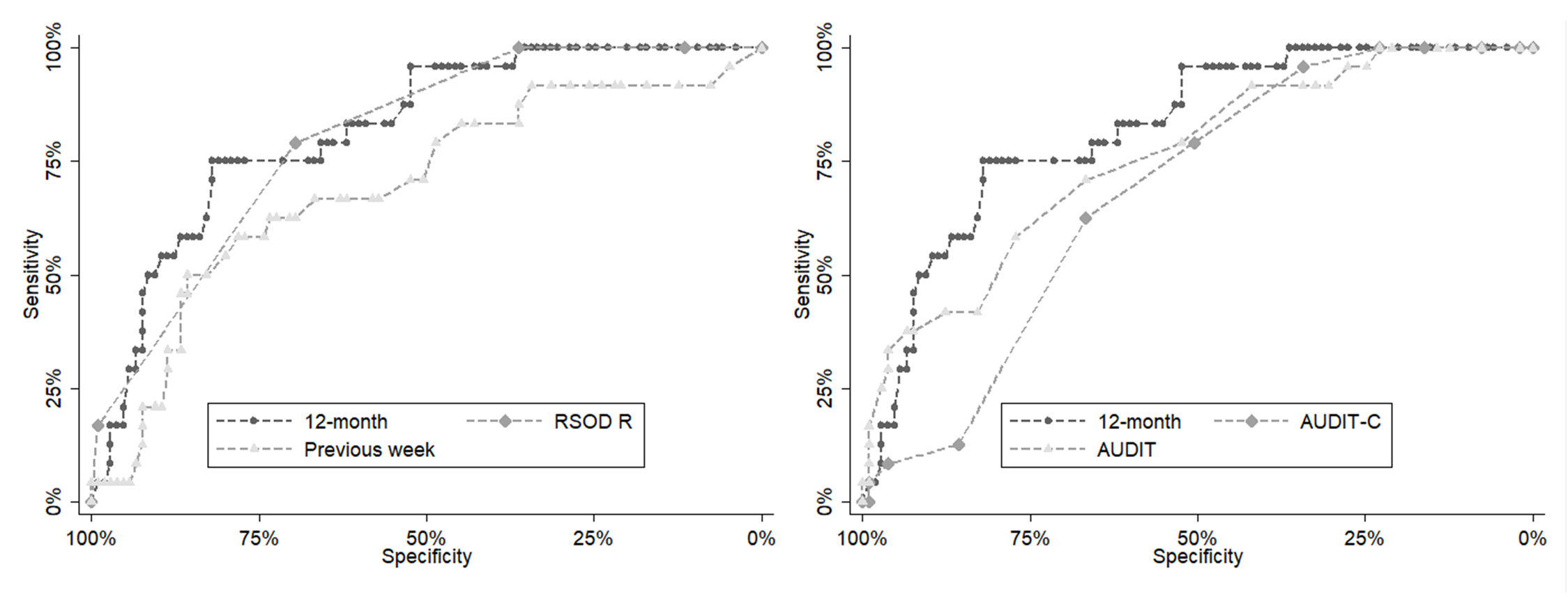

Supplement: S1 Fig — AUROC, area under the receiver operating characteristics curve for sample with hair segment between 3–6 cm. 12-month: Twelve-month alcohol use; RSOD R: Risky single-occasion drinking reversed (from ‘daily’ to ‘less than monthly’); AUDIT: Alcohol Use Disorder Identification Test; AUDIT-C: The Alcohol Use Disorders Identification Test-Consumption. (TIF) [file pone.0244336.s001.tif]
