# Supplementary material for: Performance of self-reported measures of alcohol use and of harmful drinking patterns against ethyl glucuronide hair testing among young Swiss men
Source: PLoS One. 2020 Dec 23;15(12):e0244336. doi: 10.1371/journal.pone.0244336 (PMC7757898; doi:10.1371/journal.pone.0244336)
Supplement: S1 Table — 1: mean +/- sd for quantitative variables and % (n) for categorical variables; sd: standard deviation; p50: median; p25: first quartile; p75: third quartile; RSOD: Risky single-occasion drinking; AUDIT-C: The Alcohol Use Disorders Identification Test-Consumption; AUDIT: Alcohol Use Disorder Identification Test; hEtG: ethyl glucuronide in hair. (DOCX) [file pone.0244336.s002.docx]

**S1 Table. Self-reported measures of alcohol consumption and hEtG for the whole sample and by level of hEtG for sample with hair segment between 3-6 cm.**

|  | Whole (n=129, 100%) | | | hEtG<30 (n=105, 81.4%) | | hEtG>=30 (n=24, 18.6%) | |
| --- | --- | --- | --- | --- | --- | --- | --- |
|  | [min - max] | ^1^mean +/- sd or % (n) | p50  [p25 ; p75] | ^1^mean +/- sd or % (n) | p50 [p25 ; p75] | ^1^mean +/- sd or % (n) | p50 [p25 ; p75] |
| RSOD |  |  |  |  |  |  |  |
| Daily |  | 3.9% (5) |  | 1.0% (1) |  | 16.7% (4) |  |
| Weekly |  | 35.7% (46) |  | 29.5% (31) |  | 62.5% (15) |  |
| Monthly |  | 38.8% (50) |  | 33.3% (35) |  | 20.8% (5) |  |
| Less than monthly |  | 20.2% (26) |  | 24.8% (26) |  | 0.0% (0) |  |
| Never |  | 9.3% (12) |  | 11.4% (12) |  | 0.0% (0) |  |
| Twelve-month alcohol use | [0.35 - 91] | 14.5 +/- 14.8 | 11.5 [5.5. ; 18.0] | 11.9 +/- 12.6 | 10.0 [4.0 ; 14.5] | 25.8 +/- 18.2 | 21.5 [14.8 ; 30.5] |
| Previous-week alcohol use | [0 - 153] | 20.5 +/- 19.2 | 17.0 [8.0 ; 29.0] | 18.0 +/- 15.2 | 15.0 [6.0 ; 26.0] | 31.3+/- 29.1 | 30.0 [15.0 ; 37.0] |
| AUDIT-C | [1 - 31] | 13.1 +/- 6.3 | 14.0 [9.0 ; 16.0] | 11.9 +/- 5.7 | 13.0 [8.0 ; 15.0] | 18.0 +/- 6.2 | 16.0 [14.0 ; 22.5] |
| AUDIT | [1 - 31] | 13.1 +/- 6.3 | 14.0 [9.0 ; 16.0] | 11.9 +/- 5.7 | 13.0 [8.0 ; 15.0] | 18.0 +/- 6.2 | 16.0 [14.0 ; 22.5] |
| hEtG | [0 - 191] | 19.0 +/- 28.4 | 9.3 [2.8 ; 23.0] | 9.1 +/- 7.9 | 7.3 [2.8 ; 15.0] | 62.3 +/- 42.5 | 46.0 [34.0 ; 71.5] |

^1^: mean +/- sd for quantitative variables and % (n) for categorical variables; sd: standard deviation; p50: median; p25: first quartile; p75: third quartile; RSOD: Risky single-occasion drinking; AUDIT-C: The Alcohol Use Disorders Identification Test-Consumption; AUDIT: Alcohol Use Disorder Identification Test; hEtG: ethyl glucuronide in hair.
