# Supplementary material for: Performance of self-reported measures of alcohol use and of harmful drinking patterns against ethyl glucuronide hair testing among young Swiss men
Source: PLoS One. 2020 Dec 23;15(12):e0244336. doi: 10.1371/journal.pone.0244336 (PMC7757898; doi:10.1371/journal.pone.0244336)
Supplement: S2 Table — AUROC: Area under the receiver operating characteristics curve; RSOD: Risky single-occasion drinking; AUDIT-C: The Alcohol Use Disorders Identification Test-Consumption; AUDIT: The Alcohol Use Disorders Identification Test. AUROC comparisons: 1–2: chi2(1) = 0.33, p = 0.5671; 1–3: chi2(1) = 3.93, p = 0.048; 1–4: chi2(1) = 8.83, p = 0.003; 1–5: chi2(1) = 0.73, p = 0.393; 2–3: chi2(1) = 4.43, p = 0.035; 2–4: chi2(1) = 8.08, p = 0.005; 2–5: chi2(1) = 0.98, p = 0.323; 3–4: chi2(1) = 0.0.05, p = 0.825; 3–5: chi2(1) = 0.69, p = 0.405; 4–5: chi2(1) = 2.82, p = 0.093. (DOCX) [file pone.0244336.s003.docx]

**S2 Table. The diagnostic performance of self-reported measures of acohol use in detecting heavy alcohol consumption and AUROC comparisons for sample with hair segment between 3-6 cm.**

|  | AUROC (95% CI) | Sensitivity | Specificity | Threshold |
| --- | --- | --- | --- | --- |
| RSOD (1) | 0.803 (0.727; 0.871) | 79.17% | 69.52% | weekly |
| Twelve-month alcohol use (2) | 0.820 (0.745; 0.883) | 75.00% (75.00%) | 77.14% (81.9%) | >15 (15.9) |
| Previous-week alcohol use (3) | 0.694 (0.611; 0.775) | 66.67% (66.67%) | 58.10% (66.67%) | >=19 (21) |
| AUDIT-C (4) | 0.681 (0.594; 0.761) | 62.50% | 66.67% | ≥8 |
| AUDIT (5) | 0.758 (0.677; 0.830) | 70.83% | 66.67% | ≥15 |

AUROC: Area under the receiver operating characteristics curve; RSOD: Risky single-occasion drinking; AUDIT-C: The Alcohol Use Disorders Identification Test-Consumption; AUDIT: The Alcohol Use Disorders Identification Test.

AUROC comparisons: 1-2: chi2(1) = 0.33, p = 0.5671 ; 1-3: chi2(1) = 3.93, p = 0.048; 1-4: chi2(1) = 8.83, p = 0.003 ; 1-5: chi2(1) = 0.73, p = 0.393 ; 2-3: chi2(1) = 4.43, p = 0.035; 2-4: chi2(1) = 8.08, p = 0.005 ; 2-5: chi2(1) = 0.98, p = 0.323 ; 3-4: chi2(1) = 0.0.05, p = 0.825 ;3-5: chi2(1) = 0.69, p = 0.405; 4-5: chi2(1) = 2.82, p = 0.093.
