# Supplementary material for: Performance of self-reported measures of alcohol use and of harmful drinking patterns against ethyl glucuronide hair testing among young Swiss men
Source: PLoS One. 2020 Dec 23;15(12):e0244336. doi: 10.1371/journal.pone.0244336 (PMC7757898; doi:10.1371/journal.pone.0244336)
Supplement: S3 Table — n.i.: not included; RSOD: Risky single-occasion drinking; OR: odds ratio; CI: confidence interval. (DOCX) [file pone.0244336.s004.docx]

**S3 Table. Univariate and multivariate logistic regression assessing to what extent, alcohol use and RSOD were uniquely associated with EtG for sample with hair segment between 3-6 cm.**

|  | Univariate | | | | Multivariate | | | |  |  |  |  |
| --- | --- | --- | --- | --- | --- | --- | --- | --- | --- | --- | --- | --- |
|  | OR | 95% CI | p-val | pseudo R2 | OR | 95% CI | p-val | pseudo R2 |  |  |  |  |
| RSOD | 8.67 | [2.98; 25.25 ] | <0.001 | 15.70% | 3.62 | [1.03; 12.71] | <0.001 | 21.87% |  |  |  |  |
| Twelve-month alcohol use | 10.13 | [3.61; 28.36] | <0.001 | 18.49% | 5.07 | [1.53; 16.76] | <0.001 |  |  |  |  |  |
| Previous-week alcohol use | 2.77 | [1.09; 7.05] | 0.032 | 3.92% | n.i. |  |  |  |  |  |  |  |
|  | Multivariate | | | | Multivariate | | | | Multivariate | | | |
|  | OR | 95% CI | p-val | pseudo R2 | OR | 95% CI | p-val | pseudo R2 | OR | 95% CI | p-val | pseudo R2 |
| RSOD | 10.51 | [2.80; 39.48] | <0.001 | 15.90% | n.i. |  |  | 18.49% | 4.99 | [1.18; 21.18] | 0.029 | 22.63% |
| Twelve-month alcohol use | n.i. |  |  |  | 1.00 | [0.32; 3.14] | 0.998 |  | 5.73 | [1.65; 19.91] | 0.006 |  |
| Previous-week alcohol use | 0.73 | [0.21; 2.52] | 0.617 |  | 10.13 | [3.16; 32.49] | <0.001 |  | 0.52 | [0.14; 2.00] | 0.342 |  |

n.i.: not included ; RSOD: Risky single-occasion drinking; OR: odds ratio; CI: confidence interval.
