# Supplementary material for: Performance of self-reported measures of alcohol use and of harmful drinking patterns against ethyl glucuronide hair testing among young Swiss men
Source: PLoS One. 2020 Dec 23;15(12):e0244336. doi: 10.1371/journal.pone.0244336 (PMC7757898; doi:10.1371/journal.pone.0244336)
Supplement: S2 Data — (DOCX) [file pone.0244336.s006.docx]

**AUDIT**

Because alcohol use can affect your health and can interfere with certain medications and treatments, it is important that we ask some questions about your use of alcohol, especially in the past 12 months. Your answers will remain confidential so please answer honestly. Select the option that best describes your answer to each question.

|  | **0** | **1** | **2** | **3** | **4** |
| --- | --- | --- | --- | --- | --- |
| How often do you have a drink containing alcohol? | Never | Monthly or less | 2-4 times a month | 2-3 times a week | 4 or more times a week |
| How many drinks containing alcohol do you have on a typical day when you are drinking? | 1 or 2 | 3 or 4 | 5 or 6 | 7 or 9 | 10 or more |
| How often do you have six or more drinks on one occasion? | Never | Less than monthly | Monthly | Weekly | Daily or almost daily |
| How often have you found that you were not able to stop drinking once you had started? | Never | Less than monthly | Monthly | Weekly | Daily or almost daily |
| How often have you failed to do what was normally expected of you because of drinking? | Never | Less than monthly | Monthly | Weekly | Daily or almost daily |
| How often have you needed a first drink in the morning to get yourself going after a heavy drinking session? | Never | Less than monthly | Monthly | Weekly | Daily or almost daily |
| How often have you had a feeling of guilt or remorse after drinking? | Never | Less than monthly | Monthly | Weekly | Daily or almost daily |
| How often have you been unable to remember what happened the night before because of your drinking? | Never | Less than monthly | Monthly | Weekly | Daily or almost daily |
| Have you or someone else been injured because of your drinking? | No |  | Yes, but not in the last year |  | Yes, during the last year |
| Has a relative, friend, doctor, or other health care worker been concerned about your drinking or suggested you cut down? | No |  | Yes, , but not in the last year |  | Yes, during the last year |

**Alcohol use: extended quantity-frequency questionnaire**

| How many days at weekends (from Friday to Sunday) do you drink alcohol on average? | | | | | | | | | | | | | |
| --- | --- | --- | --- | --- | --- | --- | --- | --- | --- | --- | --- | --- | --- |
|  | ❑ | 3 days in a weekend | |  | ❑ | | 2-3 weekend-days a month | | |  | |  |  |
|  | ❑ | 2 days in a weekend | |  | ❑ | | 1 weekend-day a month | | |  | |  |  |
|  | ❑ | 1 day in a weekend | |  | ❑ | | less than 1 weekend-day a month | | |  | |  |  |
|  | | | | | | ❑ | | never | | |  | |  |
| How many standard drinks do you drink on average within a weekend-day when you drink alcohol (from Friday to Sunday)? | | | | | | | | | | | | | |
|  |  |  |  |  |  | |  | |  |  | |  |  |
|  | ❑ | 12 or more | |  | ❑ | | 5 to 6 | | |  | |  |  |
|  | ❑ | 9 to 11 | |  | ❑ | | 3 to 4 | | |  | |  |  |
|  | ❑ | 7 to 8 | |  | ❑ | | 1 to 2 | | |  | |  |  |
| How many days in a week (from Monday to Thursday) do you drink alcohol on average? | | | | | | | | | | | | | |
|  | ❑ | every 4th weekdays | |  | ❑ | | 2- weekdays a month | | |  | |  |  |
|  | ❑ | 3 out of 4 weekdays | |  | ❑ | | 1 weekday a month | | |  | |  |  |
|  | ❑ | 2 out of 4 weekdays | |  | ❑ | | less than 1 weekday a month | | |  | |  |  |
|  | ❑ | 1 out of 4 weekdays | |  | ❑ | | never | | |  | |  |  |
| How many standard drinks do you have on average within a weekday (from Monday to Thursday) when you drink alcohol? | | | | | | | | | | | | | |
|  | ❑ | 12 or more | |  | ❑ | | 5 to 6 | | |  | |  |  |
|  | ❑ | 9 to 11 | |  | ❑ | | 3 to 4 | | |  | |  |  |
|  | ❑ | 7 to 8 | |  | ❑ | | 1 to 2 | | |  | |  |  |

1 standard drink


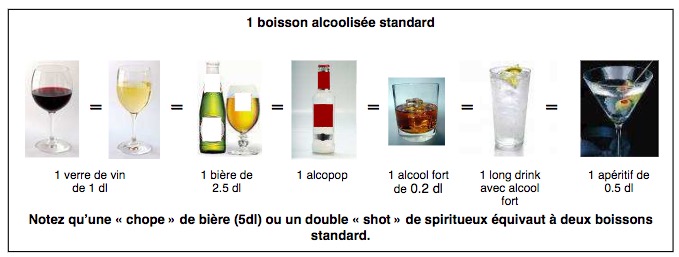


| 1 glass of wine  of 1 dl | 1 Beer  of 2.5 dl | 1 alcopop | 1 strong alcohol  of 0.2 dl | homemade Cocktail | 1 aperitif  of 0.5 dl |
| --- | --- | --- | --- | --- | --- |

**Past-week diary**

Past-week diary no. of:

|  | Beer  Nb glasses  2.5 dl | Wine (red, white, Champagne)  Nb glasses  1 dl | Strong alcohol (Whisky, Vodka, Pastis, etc)  Nb glasses  0.25 dl | Aperitif (Martini, Suze, etc)  Nb glasses  0.5 dl | Alcopops (Smirnof Ice, Bacardi Breezer, etc)  Nb bottles  3 dl | Beer pops, Wine pops, Chiller, Cooler (Cardinal Lemon, Eve, Swizly, Chiller, Strongbow, Sputnik)  Nb bottles  3 dl | Homemade Cocktail (Caipirinha,Vodka orange, Whisky Coca, etc)  Nb glasses  1 dl |
| --- | --- | --- | --- | --- | --- | --- | --- |
| Sunday |  |  |  |  |  |  |  |
| Saturday |  |  |  |  |  |  |  |
| Friday |  |  |  |  |  |  |  |
| Thursday |  |  |  |  |  |  |  |
| Wednesday |  |  |  |  |  |  |  |
| Tuesday |  |  |  |  |  |  |  |
| Monday |  |  |  |  |  |  |  |

1 standard drink


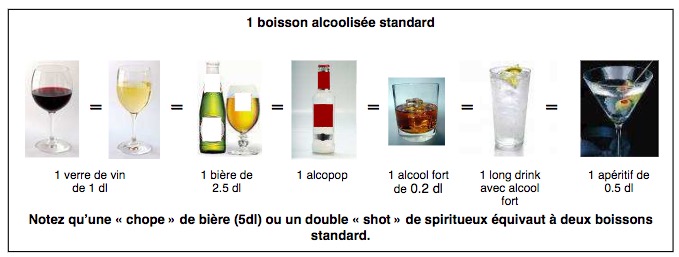


| 1 glass of wine  of 1 dl | 1 Beer  of 2.5 dl | 1 alcopop | 1 strong alcohol  of 0.2 dl | homemade Cocktail | 1 aperitif  of 0.5 dl |
| --- | --- | --- | --- | --- | --- |

**RSOD**

During the past 12 months, about how often do you drink six or more units of alcohol on a single occasion?

| ❑ | every or nearly every day |
| --- | --- |
| ❑ | every week |
| ❑ | every month |
| ❑ | less than once a month |
| ❑ | never |
